# Molecular mechanism underlying the apoptotic modulation by ethanol extract of *Pseudolarix kaempferi* in mucoepidermoid carcinoma of the salivary glands

**DOI:** 10.1186/s12935-021-02134-0

**Published:** 2021-08-14

**Authors:** Su-Jung Choi, Chi-Hyun Ahn, Kyoung-Ok Hong, Ji-Hoon Kim, Seong-Doo Hong, Ji-Ae Shin, Sung-Dae Cho

**Affiliations:** 1 Department of Oral Pathology, School of Dentistry and Dental Research Institute, Seoul National University, 03080 Seoul, Republic of Korea; 2 51-9, HLB Life Science Co., Ltd., Dongtancheomdansaneop 1-ro, 8f, Gyeonggi-do 18469 Hwaseong-si, Republic of Korea; 3 412Ho, Healthcare Innovation Park, 172 Dolma-ro, Bundang-gu, Gyeonggi-do 13605 Seongnam-si, Republic of Korea

**Keywords:** *Pseudolarix kaempferi*, Mucoepidermoid carcinoma, Mcl-1, Phosphorylation, Bcl-2, JNK signaling pathway, Apoptosis

## Abstract

**Background:**

*Pseudolarix kaempferi* is a traditional Chinese natural product that possesses the potential cytotoxic effects against cancer. However, the precise molecular mechanism underlying its cytotoxic effects has not yet been completely elucidated. Here, we clarify the mechanism via which the ethanol extract of *P*. *kaempferi* (EEPK) leads to cytotoxicity mediated by apoptosis in mucoepidermoid carcinoma (MEC) originating from the salivary glands.

**Methods:**

We investigated the mechanism underlying the anticancer efficacy of EEPK in human MEC in vitro by assessing mitochondrial dysfunction, mRNA levels, and morphological changes in apoptotic cell nuclei as well as by using a cytotoxicity assay, flow cytometric analysis, and western blotting.

**Results:**

EEPK inhibited the growth of two human MEC cells and stimulated the induction of caspase-mediated apoptosis that was accompanied by mitochondrial membrane depolarization. Compared with the vehicle control groups, EEPK decreased myeloid cell leukemia-1 (Mcl-1) expression in both cells whereas it significantly decreased B cell lymphoma-2 (Bcl-2) expression in MC3 cells only. The EEPK-induced altered Mcl-1 expression was caused by translational inhibition and proteasomal degradation. Additionally, EEPK significantly increased p-Bcl-2 (Ser^70^) expression regardless of its total forms by facilitating the activation of the c-Jun N-terminal kinase (JNK) signaling pathway, which exhibited cell context dependency. Nevertheless, JNK activation following EEPK treatment was, at least in part, required for the proapoptotic efficacy of EEPK in both cells.

**Conclusions:**

This study revealed that EEPK-induced alterations of Mcl-1 inhibition and JNK/Bcl-2 phosphorylation cause apoptosis and provided basic preclinical data for future clinical trials regarding therapy for patients with MEC.

**Graphic abstract:**

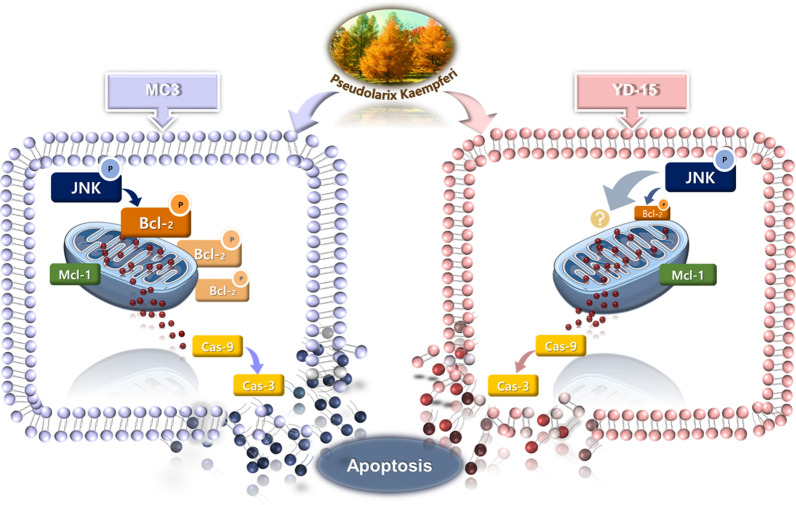

## Background

Natural products (NPs) are considered to have medicinal value because of their advantages including fewer side effects than synthetic drugs [1]. Hence, although there are numerous technical limitations in the traditional isolation process for bioactive compounds, identifying bioactive compounds for NP-based drug discovery in the pharmaceutical industry has continuously been advanced by improved scientific techniques [2]. Furthermore, the Food and Drug Administration approves NP-based anticancer drugs, and approximately 80 % of approved anticancer drugs over the past few decades have been derived from NPs and their derivatives [3]. Therefore, NPs are a promising source for discovering novel anticancer drugs and should be further explored for developing effective therapeutic strategies with improved efficiency and minimal toxicity. *Pseudolarix kaempferi* (also known as *Pseudolarix amabilis*) has been used as a traditional Chinese medicine to treat fungal skin diseases [4]. The bioactive components in *P*. *kaempferi* root bark, twigs, and seeds chiefly comprise a series of diterpenoids, triterpenoids, or lignans that display antifungal, antimicrobial, and antiviral activities [4–6]. In particular, other components isolated from *P*. *kaempferi* have been shown to exert favorable anticancer effects including *in vitro* cytotoxic properties and proapoptotic activities [7, 8]. Although accumulating evidence has shown that *P*. *kaempferi* and *P*. *kaempferi*-derived components possess anticancer properties, the precise molecular mechanism that elicits the anticancer effects of *P*. *kaempferi* remains unclear.

B cell lymphoma-2 (Bcl-2) family proteins are classified into three subgroups based on their function and the presence of Bcl-2 homology (BH) domains (e.g., antiapoptotic and proapoptotic multidomains and proapoptotic BH3-only proteins). Bcl-2 proteins control the balance between life and death of cells by regulating mitochondrial outer membrane permeabilization, following which mitochondrial intermembrane space proteins, such as cytochrome c and Smac, are released from mitochondria, thereby inducing caspase-mediated apoptosis [9]. Particularly, antiapoptotic Bcl-2 family proteins [e.g., Bcl-2, Bcl-xL, myeloid cell leukemia-1 (Mcl-1), and Bcl-W] protect cells from apoptosis by heterodimerizing with the proapoptotic Bcl-2 family protein Bax/Bak until being neutralized by BH3-only proteins; this phenomenon is associated with carcinogenesis and poor susceptibility to chemotherapy [10]. These proteins share structural homology in four conserved BH domains, and targeting the BH3 or BH4 domains is considered as a promising strategy for cancer therapy [11, 12]. In particular, some agents derived from naturally occurring compounds that target antiapoptotic Bcl-2 family proteins have been isolated and synthesized to identify further potent Bcl-2 inhibitors [13, 14]. Thus, discovering potential therapeutic inhibitors derived from naturally occurring compounds that target antiapoptotic Bcl-2 family proteins might provide an effective therapeutic strategy for patients with cancer.

In the present study, we examined the molecular mechanism underlying the apoptotic cytotoxicity following treatment with the ethanol extract of *P*. *kaempferi* (EEPK), which was associated with the Mcl-1 inhibition in human mucoepidermoid carcinoma (MEC) cells caused by translation inhibition and proteasome-mediated degradation. Additionally, EEPK stimulated the phosphorylation of Bcl-2 at the Ser^70^ residue by activating the c-Jun N-terminal kinase (JNK) signaling pathway in a cell context-dependent manner. Overall, these results supported the possibility that EEPK is a promising therapeutic agent with proapoptotic efficacy for treating patients with MEC.

## Methods

### Preparation of ethanol extract

The root bark of *P*. *kaempferi* (553 g) was purchased from a herb store (Wonju, Republic of Korea) and extracted three times with ethanol (EtOH), under reflux, for 5 h. The extracted solution was filtered and evaporated under reduced pressure on a rotatory evaporator to produce 18.6 g of EtOH extract. To fractionate the EtOH extract, it was suspended in H_2_O and fractionated three times with hexane. The residual aqueous layer was further fractionated three times with EtOAc. The EtOAc-soluble portion was concentrated to dryness to yield the EtOAc extract (5.78 g), which was dissolved in dimethyl sulfoxide (DMSO), aliquoted, and stored at − 20 °C. The final concentration of DMSO did not exceed 0.1 %.

### Cell culture and reagents

Two cells of MEC originating from the salivary glands were used. MC3 cells were kindly provided by Prof. Wu Junzheng from the Fourth Military Medical University (Xi’an, China), and YD-15 cells were obtained from the Korean Cell Line Bank (Seoul, Republic of Korea). The cells were grown in DMEM/F12 or RPMI 1640 medium supplemented with 10 % fetal bovine serum and 1 % penicillin/streptomycin in a humidified atmosphere incubator containing 5 % CO_2_ at 37 °C. All experiments were performed on cells cultured to 50–60 % confluence. All reagents are listed in Additional file 1: Table S1.

### Trypan blue exclusion assay

The cells were seeded in 6-well plates and incubated with various doses of EEPK for 24 h. Following cell detachment by trypsinization, the cells were stained with 0.4 % trypan blue solution; thereafter the viable cells were counted using a hemocytometer.

### Cell counting kit-8 (CCK-8) assay

Cells were seeded in 96-well plates and incubated with various doses of EEPK for 24 h. Subsequently, 10 µl of the CCK-8 solution was directly added to each well and mixed thoroughly with the culture media. Following incubation at 37 °C for 2 h, the optical density of each well was measured using the Chameleon microplate reader (Hidex, Turku, Finland) at 450 nm.

### DAPI staining

After being subjected to the designated treatment, the cells were fixed with 100 % EtOH at − 20 °C overnight and 100 % MeOH at room temperature for 10 min. Thereafter, the fixed cells were placed onto slides and stained with 2 µg/ml of DAPI solution. The morphological changes of the cells were observed under a fluorescence microscope (Leica DMi8; Leica Microsystems GmbH, Wetzlar, Germany).

### Annexin V-FITC/PI double staining

To assess cells undergoing the early or late stages of apoptosis, the FITC-Annexin V Apoptosis Detection Kit was used according to the manufacturer’s instructions. The stained cells were analyzed using the FACSCalibur Flow Cytometer (BD Biosciences, San Jose, CA, USA) and the proportion of stained cells was quantified from 10,000 cells per sample with BD CellQuest™ Pro software. Annexin V^+^/PI^−^ staining indicates the early stage of apoptosis, whereas Annexin V^+^/PI^+^ staining indicates the late stage of apoptosis. The flow cytometry data were reanalyzed using FlowJo software version 9/10 (FlowJo LLC, Ashland, OR, USA).

### Analysis of the sub-G_1_ population

Cells were fixed with 70 % EtOH overnight at − 20 °C followed by incubation with 20 µg/ml of RNase A and propidium iodide solution for 15 min at 37 °C. The cell cycle distribution was detected using the FACSCalibur Flow Cytometer, and the proportion of stained cells was quantified from 10,000 cells per sample with BD CellQuest™ Pro software. The relative percentages of each phase were reanalyzed using FlowJo software version 9/10.

### Western blotting

Total proteins were extracted with RIPA lysis buffers including phosphatase inhibitor tablets and protease inhibitor cocktails. The protein concentrations in each sample were measured using the *DC* Protein Assay Kit. Following normalization, equal amounts of protein were separated using SDS-PAGE and then transferred to Immun-Blot^®^ PVDF membranes. The membranes were blocked using 5 % skim milk in TBS with Tween 20 at room temperature for 2 h. Thereafter, they were incubated overnight at 4 °C with primary antibodies. Subsequently, the membranes with primary antibodies were incubated with HRP-conjugated secondary antibodies for 2 h at room temperature. The antibodies of all target proteins are provided in Additional file 1: Table S2. The immunoreactive bands were visualized using WestGlow™ PICO PLUS Chemiluminescent substrate followed by ImageQuant™ LAS 500 (GE Healthcare Life Sciences, Piscataway, NJ, USA) or x-ray film.

### Mitochondrial membrane potential (ΔΨm) assay

ΔΨm was measured by flow cytometry using the lipophilic fluorescent dye JC-1. Cells were harvested via trypsinization, washed with PBS, and pelleted via centrifugation at 3500 rpm for 5 min. The pellets were resuspended in a 1× JC-1 working solution and incubated at 37 °C for 30 min in the dark. The stained cells were washed with a 1× assay buffer and pelleted via centrifugation at 3500 rpm for 5 min. After removing the supernatant, the cells were resuspended in a 1× assay buffer. Subsequently, the cells were transferred to FACS tubes and analyzed with the FACSCalibur Flow Cytometer. The proportion of stained cells was quantified from 10,000 cells per sample with BD CellQuest™ Pro software. The data were reanalyzed using FlowJo software.

### Cytosolic and mitochondrial fractions

Cytosolic and mitochondrial fractions were isolated with the Mitochondria/Cytosol Fractionation Kit. Briefly, cells were washed with ice-cold PBS, and the cell pellet was resuspended in a 1× cytosol extraction buffer mix containing DTT and protease inhibitor for 10 min on ice. Following centrifugation at 13,000 rpm for 15 min at 4 °C, the supernatants containing cytosolic proteins were collected, and the pellets were resuspended in a mitochondrial extraction buffer mix. The supernatant containing mitochondrial proteins was collected from the final centrifugation.

### Quantitative real-time PCR (qPCR)

Total RNA was extracted using TRIzol Reagent. Total RNA (1 µg) was reverse-transcribed using the AMPIGENE cDNA Synthesis Kit; the resultant cDNA was subjected to PCR using the AMPIGENE qPCR Green Mix Hi-Rox. The qPCR instrument was assembled using the Applied Biosystems StepOnePlus Real-Time PCR System (Applied Biosystems, Foster City, CA, USA), and PCR conditions for all genes were as follows: 95 °C for 2 min, followed by 40 cycles of 95 °C for 10 s and 60 °C for 30 s. The relative amount of *Mcl-1* expression was normalized to the amount of *GAPDH* expression and calculated using the 2^−∆∆Ct^ method. The qPCR primers of all the target genes are listed in Additional file 1: Table 3.

### Statistical analysis

All data were transformed using GraphPad Prism version 7.0 (La Jolla, CA, USA). Statistical analyses were performed using SPSS version 22.0 (SPSS Inc., Chicago, IL, USA). Two-tailed Student’s t-tests for two comparisons or one-way ANOVA with Tukey’s post hoc test for multiple comparisons were used; *p* values of < 0.05 were considered statistically significant.

## Results

### EEPK inhibits the growth and stimulates the induction of apoptosis in human MEC cells

**Fig. 1:**
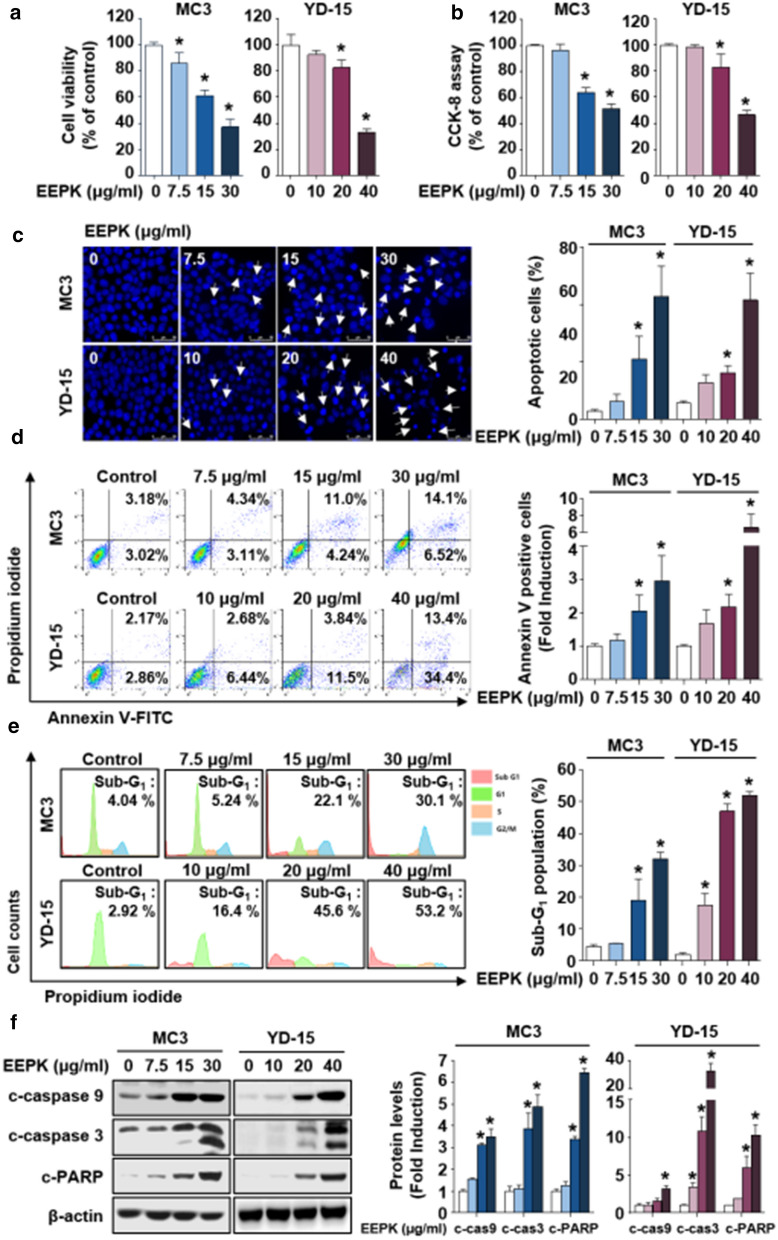
Cytotoxic and apoptotic effects of EEPK on human MEC cells. MC3 and YD-15 cells were treated with DMSO or various doses of EEPK (7.5, 15, and 30 µg/ml for MC3 and 10, 20, and 40 µg/ml for YD-15) for 24 h. The cytotoxic effect of EEPK was determined by trypan blue exclusion assay (**a**) and CCK-8 assay (**b**). **c** Representative images of DAPI-stained human MEC cells (magnification, 400×). The white arrows indicate apoptotic cells. Scale bar, 50 μm. **d** Annexin V/PI double staining was assessed using FACS analysis. **e** The population of sub-G_1_ in human MEC cells with or without EEPK. **f** Western blot images showing the expression levels of c-caspase 9, c-caspase 3, and c-PARP. β-actin was used as an internal control. All bar graphs represent the mean ± SD of three independent experiments. ^*^p < 0.05

To ascertain the potentially cytotoxic effect of EEPK on human MEC cells, we first conducted dose–response tests using a trypan blue exclusion assay and CCK-8 assay after treatment with various doses of EEPK. It was observed that EEPK significantly decreased the growth of two human MEC cells in a dose-dependent manner (Fig. 1a, b). To determine whether the cytotoxic effect of EEPK on human MEC cells was related to apoptosis, the cells were stained with DAPI solution to detect chromatin condensation and DNA fragmentation, the typical morphological characteristics of apoptotic cells. Abundant visualization of distinctively compacted chromosome or DNA fragments was achieved following EEPK treatment (Fig. 1c). We further investigated the apoptotic effect of EEPK using flow cytometric analysis. Compared with the vehicle control groups, the rate of Annexin V^+^ cells undergoing early-stage (Annexin V^+^/PI^−^) or late-stage (Annexin V^+^/PI^+^) apoptosis was apparently increased following EEPK treatment (Fig. 1d). Accordingly, a significant accumulation of apoptotic cells in the sub-G_1_ phase was observed in the EEPK-treated cells (Fig. 1e). To further corroborate EEPK-induced apoptosis, we evaluated the expression of apoptosis marker proteins via western blotting and observed an abundant cleavage of caspase 9, caspase 3, and PARP in a dose-dependent manner following EEPK treatment (Fig. 1f). Collectively, these results indicated that EEPK inhibits the growth and stimulates the induction of apoptosis via a caspase-mediated pathway in human MEC cells.

### **EEPK decreases Mcl-1 and Bcl-2 protein levels to facilitate apoptosis following mitochondrial membrane depolarization**

**Fig. 2:**
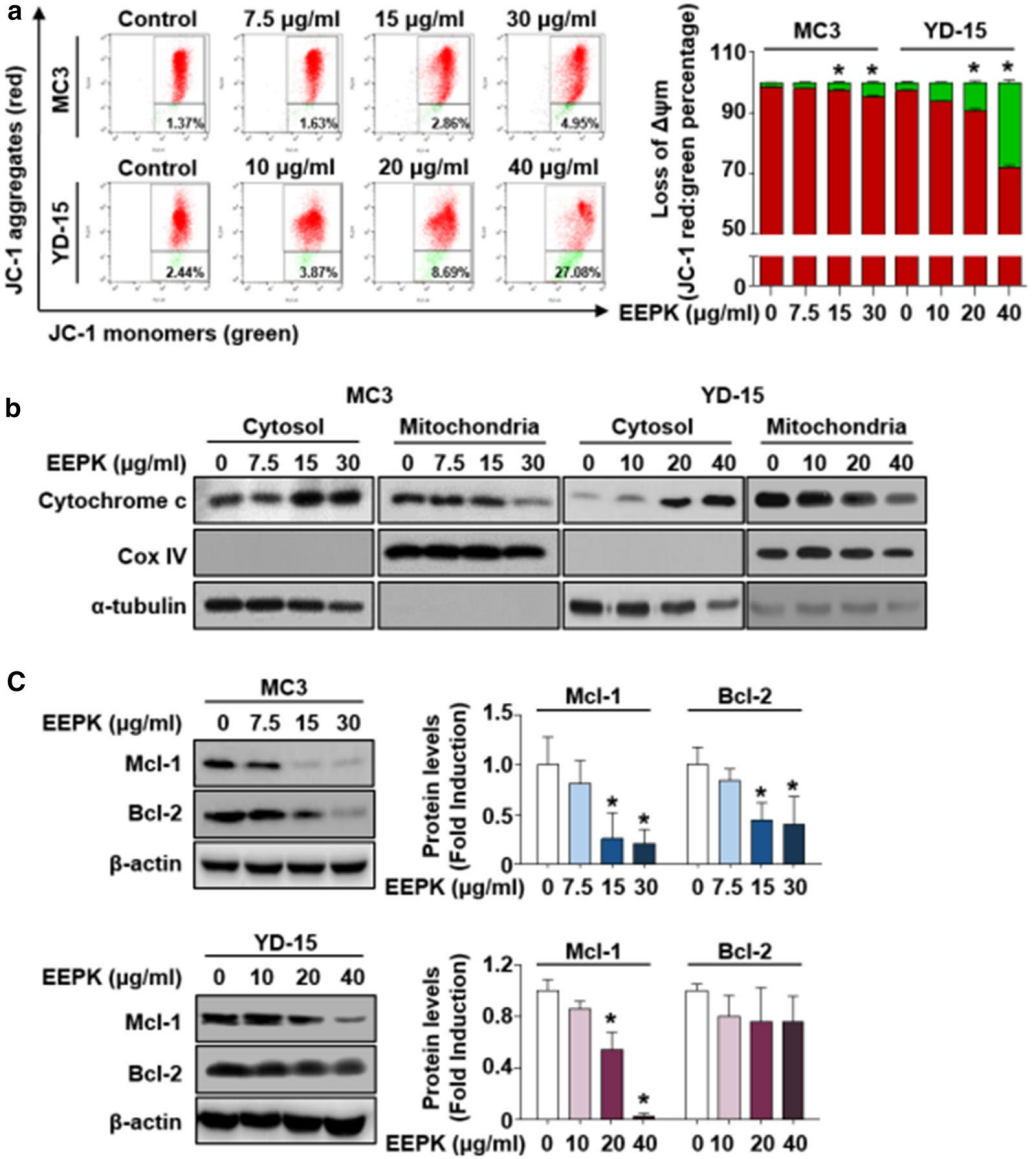
Effect of EEPK on disrupted mitochondrial membrane potential, cytochrome c release, and antiapoptotic Bcl-2 family proteins. MC3 and YD-15 cells were treated with DMSO or the indicated doses of EEPK for 24 h. **a** Measurement of mitochondrial membrane potential in human MEC cells with or without EEPK using a JC-1 probe. Bar graphs represent the mean ± SD of three independent experiments. ^*^p < 0.05. **b** Cytochrome *c* release into the cytosol was evaluated using cytosolic and mitochondrial fractions. Cox IV and α-tubulin were used as specific fraction markers for mitochondria and cytosol, respectively. Data are representative of two independent experiments. **c** Western blot images showing the expression levels of Mcl-1 and Bcl-2. β-actin was used as an internal control. All bar graphs represent the mean ± SD of three independent experiments. ^*^p < 0.05

To investigate whether the EEPK-induced apoptosis in human MEC cells was accompanied by mitochondrial dysfunction, the cationic dye JC-1 that can detect changes in mitochondrial membrane potential was used. Compared with the vehicle control groups, the monomeric form of JC-1 (cytosolic green fluorescence) was increased in the EEPK-treated groups (Fig. 2a), implying a disrupted mitochondrial membrane potential. Additionally, EEPK caused the release of cytochrome c from the mitochondria into the cytosol (Fig. 2b). To determine the key molecules underlying mitochondrial membrane depolarization following EEPK treatment, we detected antiapoptotic Bcl-2 family proteins, which regulate mitochondrial dysfunction followed by the induction of apoptosis. Significantly decreased Mcl-1 expression was observed in two human MEC cells, whereas significantly decreased Bcl-2 expression was observed in only the MC3 cells (Fig. 2c). Conversely, EEPK showed no apparent effect on Bcl-xL expression in both cells (Additional file 1: Fig. S1). Taken together, these results indicated that EEPK results in mitochondrial membrane depolarization and cytochrome c release accompanied by the decreased expression of negative regulators of apoptosis, such as Mcl-1 and Bcl-2 proteins, in human MEC cells.

### **Mcl-1 downregulation induced by EEPK is accomplished by translation inhibition and proteasome-mediated degradation**

**Fig. 3:**
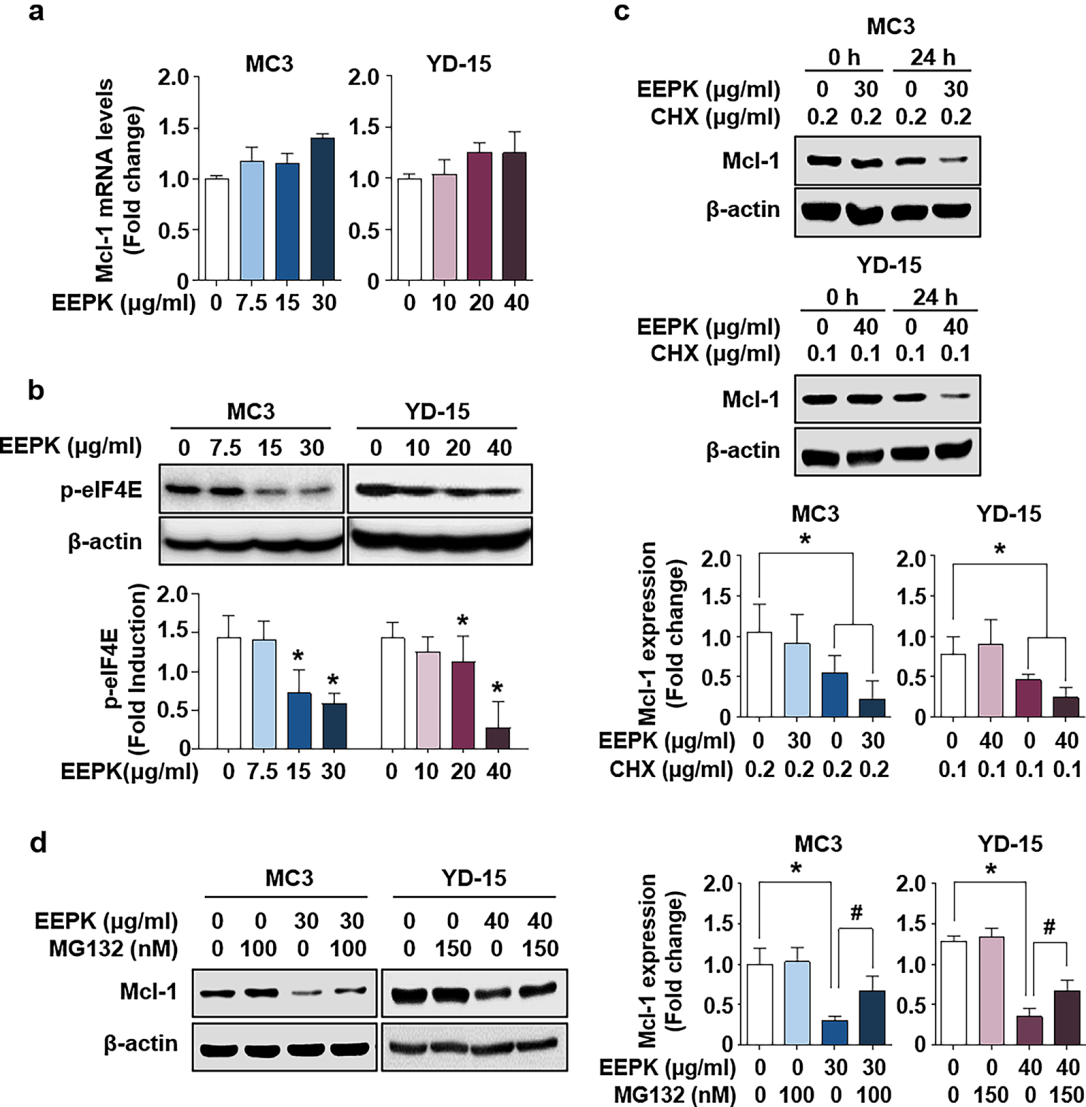
Effect of EEPK on Mcl-1 protein turnover in human MEC cells. **a** MC3 and YD-15 cells were treated with DMSO or the indicated doses of EEPK for 24 h. Relative mRNA levels of *Mcl-1* in EEPK-induced human MEC cells were assessed using qPCR and normalized to *GAPDH*. Data are shown as the mean ± SD of three independent experiments. **b** The protein levels of p-eIF4E were examined with western blotting. β-actin was used as an internal control. All bar graphs represent the mean ± SD of three independent experiments. ^*^p < 0.05. **c** Both cells were pretreated with the indicated doses of CHX for 1 h in the absence or presence of EEPK for 24 h. Representative western blot images for Mcl-1 expression. **d** Pretreatment with the indicated doses of MG132 for 1 h in the absence or presence of EEPK for 24 h. The protein levels of Mcl-1 were determined via western blotting. β-actin was used as an internal control. All bar graphs represent the mean ± SD of three independent experiments. ^*^p < 0.05; ^#^*p* < 0.05

To examine any dose-dependent effect of EEPK on Mcl-1 mRNA levels in human MEC cells, we performed qPCR. EEPK had no significant effect on Mcl-1 mRNA levels in either cell line (Fig. 3a), indicating the possibility of translational or posttranslational regulation of Mcl-1. To clarify whether the decrease in Mcl-1 protein levels induced by EEPK treatment was caused by its translational inhibition, we examined the phosphorylation status of eIF4E, a translation initiation factor. As shown in Fig. 3b, compared with the vehicle control groups, EEPK resulted in an apparent decrease of p-eIF4E expression in both cells. To further investigate whether EEPK could regulate the stability of Mcl-1, we used cycloheximide (CHX) to block new protein synthesis. Mcl-1 expression in the EEPK- and CHX-treated groups was more reduced than in the CHX-treated groups (Fig. 3c). Furthermore, pretreatment with MG132, a potent proteasome inhibitor, rescued the EEPK-induced decrease in Mcl-1 expression (Fig. 3d). These results indicated that EEPK facilitates the decrease in Mcl-1 protein levels via the induction of translational inhibition and proteasome-mediated degradation, suggesting that Mcl-1 downregulation via translation inhibition and proteasomal degradation is an important event for apoptosis of human MEC cells following EEPK treatment.

### **EEPK induces phosphorylated Bcl-2 (Ser**^**70**^**) by activating the JNK signaling pathway**

**Fig. 4:**
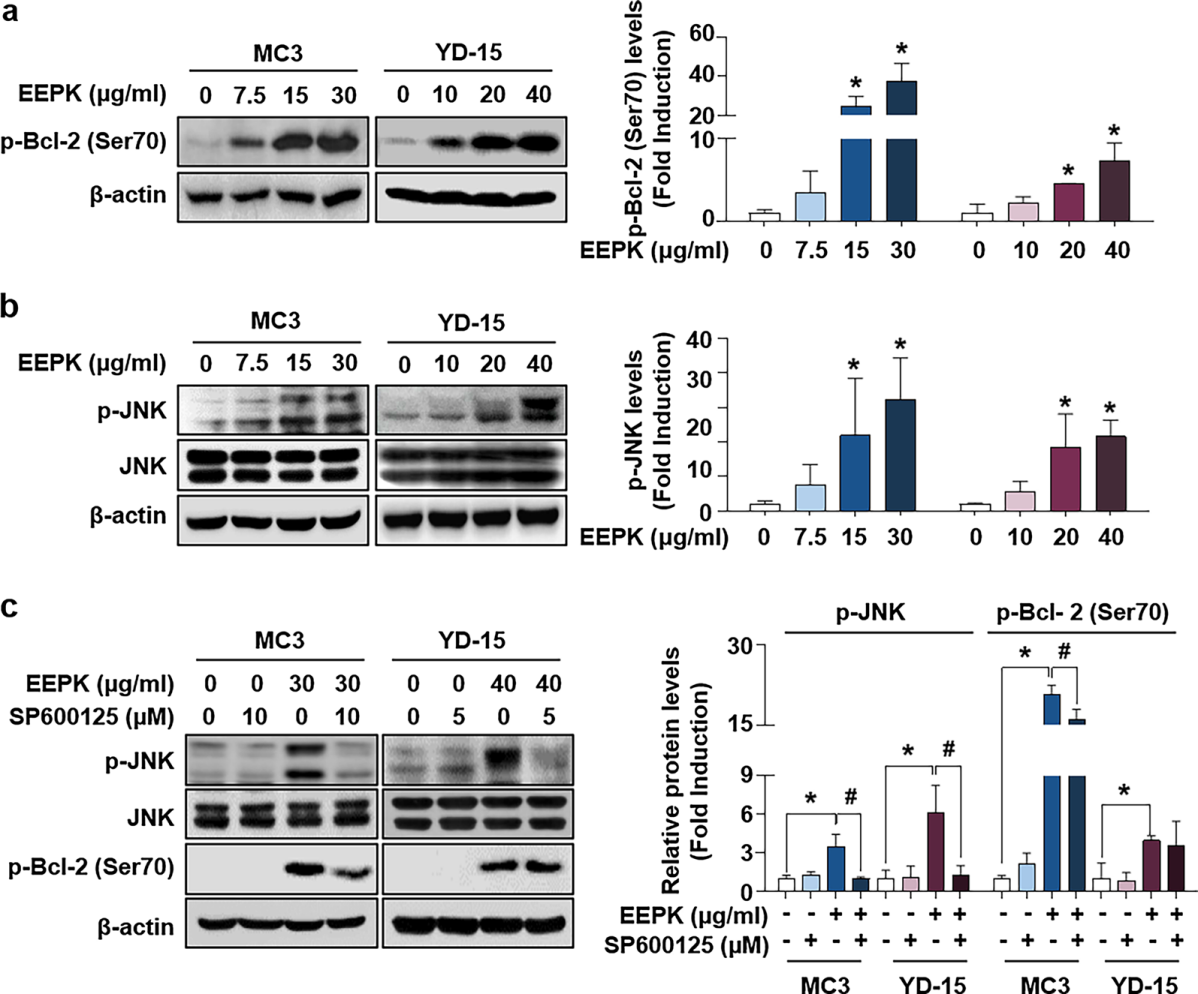
Effect of EEPK on phosphorylated Bcl-2 (Ser^70^) through the activation of the JNK signaling pathway. MC3 and YD-15 cells were treated with DMSO or indicated doses of EEPK for 24 h. **a** Western blot images showing p-Bcl-2 (Ser^70^) expression levels. **b** The p-JNK and JNK expression levels were analyzed with western blotting. β-actin was used as an internal control. **c** Both cells were pretreated with the indicated doses of SP600125 for 1 h and then treated with DMSO or EEPK for 24 h. Representative western blot images showing the expression levels of p-JNK, JNK, and p-Bcl-2 (Ser^70^). β-actin was used as an internal control. All bar graphs represent the mean ± SD of three independent experiments. ^*^p < 0.05; ^#^*p* < 0.05

To examine the contribution of EEPK to Bcl-2 phosphorylation, we evaluated Bcl-2 phosphorylation at the serine 70 residue in human MEC cells following EEPK treatment. EEPK significantly increased p-Bcl-2 (Ser^70^) expression in both cells compared with that in the vehicle control groups (Fig. 4a). To investigate the involvement of the mitogen-activated protein kinase signaling pathway in EEPK-induced Bcl-2 phosphorylation, the phosphorylated forms of JNK, p38, and ERK1/2 in both cells following EEPK treatment were detected with their corresponding total forms. Compared with p-JNK expression levels in the vehicle control groups, those after EEPK treatment were consistently induced in both cells in which the basal levels of total JNK remained unchanged (Fig. 4b). Conversely, no consistent dose-dependent effect on the expression levels of p-p38 or p-ERK1/2 was observed in either cell line following EEPK treatment (Additional file 1: Fig. S2). To elucidate the role of JNK activation on EEPK-induced Bcl-2 phosphorylation, we used the potent reversible JNK inhibitor SP600125. Compared with EEPK treatment alone, the addition of SP600125 before EEPK treatment modestly abolished Bcl-2 phosphorylation at the Ser^70^ residue only in the MC3 cell line (Fig. 4c). Collectively, these data suggested that JNK activation following EEPK treatment is required for Bcl-2 phosphorylation at the Ser^70^ residue in human MEC cells in a cell context-dependent manner.

### JNK activation is responsible for EEPK-induced apoptosis

**Fig. 5:**
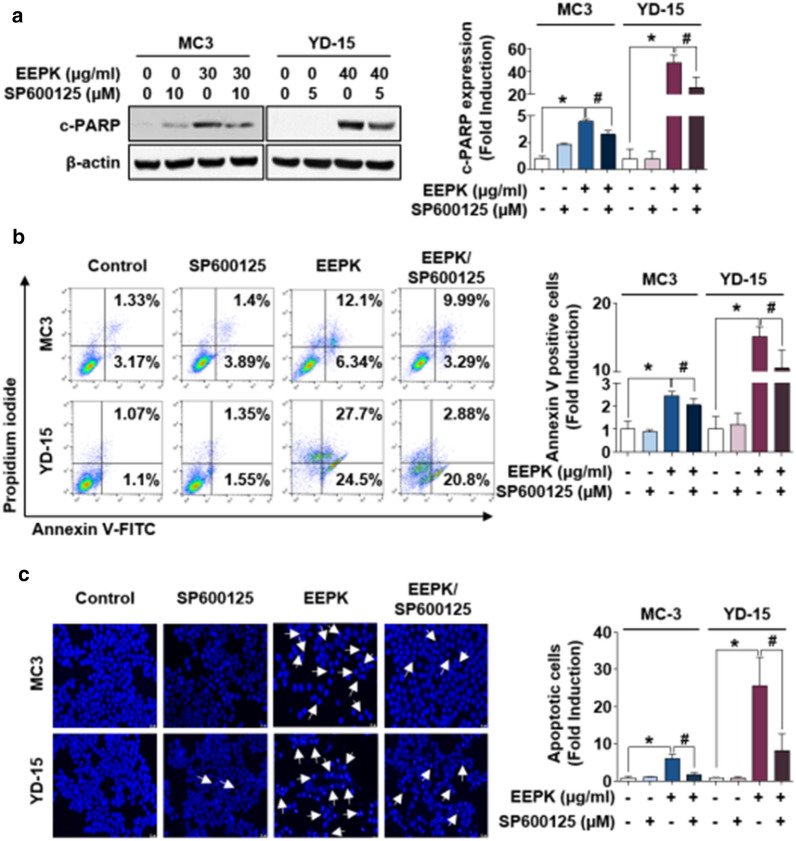
Contribution of JNK activation on apoptosis induced by EEPK. MC3 and YD-15 cells were pretreated with the indicated doses of SP600125 for 1 h and then treated with DMSO or EEPK for 24 h. **a** Western blotting was performed to detect the expression levels of cleaved PARP. β-actin was used as an internal control. **b** Annexin V/PI double staining was measured by FACS analysis. **c** Representative images of DAPI-stained cells (magnification, 400×). The white arrows indicate apoptotic cells. Scale bar, 20 μm. All bar graphs represent the mean ± SD of three independent experiments. ^*^p < 0.05; ^#^*p* < 0.05

To define the contribution of JNK activation on EEPK-induced apoptosis, MC3 and YD-15 cells were pretreated with SP600125 to detect altered PARP cleavage. Following EEPK treatment, SP600125 partly abrogated the abundant cleavage of PARP in both cells (Fig. 5a) and reduced the proportion of apoptotic cells (Fig. 5b). Furthermore, compared with EEPK treatment alone, cotreatment with SP600125 displayed less compacted chromosome or DNA fragments (Fig. 5c). Taken together, these data suggested that JNK activation can be a critical event for modulating apoptosis in human MEC cells following EEPK treatment.

## Discussion

MEC is the most commonly diagnosed tumor of the salivary glands and is composed of three different cell types: mucinous, epidermoid, and intermediated cells [15]. Typically, in cases of salivary gland tumors, severe side effects can occur postoperatively, including permanent facial nerve paralysis from infiltration of the facial nerve and varying degrees of xerostomia [16, 17]. Furthermore, it is extremely difficult to predict the disease progression because of its anatomically complex and varied pathological forms. To solve this issue, studies have investigated novel approaches for the diagnosis and treatment of salivary gland tumors (that have several genetic alterations); however, there have been no notable achievements. Therefore, novel and effective therapeutic options for patients with MEC are needed. In the present study, we provided a promising chemotherapeutic option for patients with MEC by demonstrating the apoptosis induced by EEPK and its predicted oncological targets.

Mcl-1, a member of the antiapoptotic Bcl-2 protein family, is highly expressed in various tumor types and is associated with poor prognoses and poor clinical outcomes owing to tumor resistance to anticancer drugs [18]. In response to various extracellular stimuli including cytokines and growth factors, Mcl-1 expression levels are thoroughly controlled via multiple processes such as transcriptional, posttranscriptional, and posttranslational regulation [19], thereby determining cancer cell survival or death. In the present study, we observed that Mcl-1 inhibition in human MEC cells following EEPK treatment was not caused by altered Mcl-1 mRNA levels. Therefore, we excluded the possibility that Mcl-1 expression was regulated at the transcriptional level upon EEPK treatment. Previous studies have reported that translational inhibition of Mcl-1 expression in response to naturally occurring compounds attributes to the induction of G_2_/M cell cycle arrest or apoptosis in several cancer cells, which was involved in the phosphorylation status of translation initiation factors such as eIF4E or eIF4G [20, 21]. In the present study, we observed an apparent decrease in the phosphorylated levels of eIF4E, suggesting that the EEPK-induced inhibition of eIF4E phosphorylation contributes to the decrease in Mcl-1 protein levels. Reportedly, the posttranslational modification of Mcl-1 is accompanied by ubiquitination, cleavage, and phosphorylation, which decides the protein turnover, localization, and function [22]. In particular, our previous findings have shown that Mcl-1 inhibition induced by naturally occurring compounds in oral cancer cells is involved in lysosome- or proteasome-dependent degradation [23, 24]. These results led us to speculate that EEPK may cause a rapid turnover of Mcl-1 protein in human MEC cells. The results from our present study have clearly revealed that MG132-induced proteasomal inhibition restored the EEPK-induced decrease in Mcl-1 expression. Therefore, the EEPK-induced Mcl-1 reduction was clearly caused by translation inhibition and proteasome-dependent degradation rather than by changing mRNA levels. It was previously suggested that the rapid turnover of Mcl-1 protein depends on its phosphorylation by several kinases including GSK3, which is closely related to the activity of E3 ubiquitin ligases [25, 26]. Thus, future studies should focus on identifying the upstream target molecules (e.g., kinases or E3 ubiquitin ligases) that result in a rapid turnover of Mcl-1 protein in human MEC cells.

A previous study reported that medicinal plant extracts, such as those of *Vernonia condensata* contribute toward triggering intrinsic apoptotic pathway, as indicated by disruption of mitochondrial membrane potential and induction of proapoptotic Bcl-2 family protein [27]. In the present study, we observed that EEPK had no effect on Bcl-2 expression in YD-15 cells, despite the decreased Bcl-2 expression in MC3 cells. It has been reported that the ability of Bcl-2 to protect cells from apoptosis could be regulated by its posttranslational modification, such as phosphorylation, occurring in four serine/threonine residues (Thr^56^, Thr^69^, Ser^70^, and Ser^87^) within the loop domain [28, 29]. Bcl-2 protein phosphorylation triggers the inactivation and degradation of the protein [30, 31]. Although speculations regarding the biological function of Bcl-2 phosphorylation have been controversial, it is certain that Bcl-2 phosphorylation at the Ser^70^ residue is important and frequently occurs in several cancer types following exposure to microtubule-targeting drugs [32]. In particular, patients with colorectal adenocarcinoma without p-Bcl-2 (Ser^70^) expression exhibit shorter survival rates than patients with positive p-Bcl-2 (Ser^70^) expression, which is correlated with lymph node metastasis and clinical stages [33]. Considering the critical role of Bcl-2 phosphorylation at the Ser^70^ residue, we investigated p-Bcl-2 (Ser^70^) expression levels induced by EEPK in human MEC cells. The results clearly showed that EEPK increased p-Bcl-2 (Ser^70^) expression levels in both cells in a dose-dependent manner unlike the cell context-dependent response of total Bcl-2 levels. A previous study reported that Bcl-2 phosphorylation disrupts the interaction between Bcl-2 and Bax, facilitating Bax release and the subsequent induction of apoptosis [34]. Therefore, we cautiously speculated that the increased p-Bcl-2 (Ser^70^) expression in human MEC cells following EEPK treatment may primarily contribute to the induction of apoptosis by terminating interactions between p-Bcl-2 and its binding partners, such as the proapoptotic Bcl-2 family protein Bax, rather than by decreasing total Bcl-2 protein. Additional studies are required to elucidate the interactions of Bcl-2 binding partners.

Among the three mitogen-activated protein kinases, JNK plays a key role in facilitating the apoptosis signaling pathway in response to both extracellular and intracellular stress stimuli [35]. JNK phosphorylation at residues Thr183 and Tyr185 induced by two upstream mitogen-activated protein kinase kinase typically regulates the activation of specific downstream target molecules; this is responsible for various cellular activities, including apoptosis, proliferation, migration, survival, differentiation, and inflammation [36]. It was previously reported that the activation of the JNK signaling pathway is sufficient to phosphorylate Bcl-2 at both residues Ser^70^ and Thr^56^, thereby inducing apoptosis in prostate cancer *in vitro* [37–39]. In the present study, we observed that EEPK stimulated the activation of the JNK signaling pathway in both cells in a dose-dependent manner, indicating that the activated JNK signaling pathway participates in Bcl-2 phosphorylation. Further, we investigated the involvement of the JNK signaling pathway on Bcl-2 phosphorylation at the Ser^70^ residue. SP600125, a potent reversible inhibitor of JNK, suppressed p-Bcl-2 (Ser^70^) expression levels in EEPK-treated MC3 cells. However, Bcl-2 phosphorylation in EEPK-treated YD-15 cells was not influenced by SP600125, although the activated JNK signaling pathway was completely inhibited. Remarkably, SP600125 significantly abrogated EEPK-induced apoptosis in both MC3 and YD-15 cells regardless of p-Bcl-2 expression. It appears that EEPK-induced Bcl-2 phosphorylation may be one of the molecular mechanisms leading to apoptosis in human MEC cells. As described above, the activation of the JNK signaling pathway contributes to the phosphorylation of several downstream targets including c-Jun, ATF, and STAT3 [36]. Therefore, we presumed that JNK activation might regulate other downstream targets, thereby stimulating proapoptotic activity in human MEC cells; these targets should be investigated in the future. Nevertheless, we believe that our data revealed the basic molecular mechanism via which EEPK, at least in part, phosphorylates Bcl-2 protein at the Ser^70^ residue; namely, by activating the JNK signaling pathway and thereby inducing apoptosis in human MEC cells.

## Conclusions

The results of the present study demonstrated that EEPK promotes apoptosis by targeting Mcl-1 and the JNK/Bcl-2 signaling pathway, which, in turn, inhibits the growth of human MEC cells. These findings provide molecular insight into the potential anticancer efficacy of EEPK, thereby contributing to the development of future human MEC therapeutics.

## Data Availability

The datasets used and/or analyzed during the current study are available from the corresponding author upon reasonable request.
